# Supplementary material for: Half-duplex and full-duplex interference mitigation in relays assisted heterogeneous network
Source: PLoS One. 2023 Jun 20;18(6):e0286970. doi: 10.1371/journal.pone.0286970 (PMC10281590; doi:10.1371/journal.pone.0286970)
Supplement: Attachment — Submitted filename: Response_to_Reviewers.pdf [file pone.0286970.s001.pdf]

Manuscript ID PONE-D-22-34295

Firstly, we would like to thank the editor and the reviewers for their efforts in reviewing our manuscript. The comments are useful and constructive. Following the comments, we have made changes to improve the final manuscript. We also respond to individual concerns of the reviewers, where the comments are printed in italic, followed by the responses. In the revised manuscript, all changes are indicated by red colored fonts. The manuscript is edited in order to comply with length policy imposed by PLOS ONE Journal.

Response to Editor's Comments

We have ensured that the edited manuscript version meets PLOS ONE's style requirements, including those for file naming. The paper-generated code is made available without restrictions on https://github.com/MoubachirMadani/MIMO_Half-Duplex_Full-Duplex_Relay.

Reviewers' comments:

1. Is the manuscript technically sound, and do the data support the conclusions?

The manuscript must describe a technically sound piece of scientific research with data that supports the conclusions. Experiments must have been conducted rigorously, with appropriate controls, replication, and sample sizes. The conclusions must be drawn appropriately based on the data presented.

Reviewer #1: Yes

Reviewer #2: Yes

2. Has the statistical analysis been performed appropriately and rigorously?

Reviewer #1: Yes

Reviewer #2: No

3. Have the authors made all data underlying the findings in their manuscript fully available?

The PLOS Data policy requires authors to make all data underlying the findings described in their manuscript fully available without restriction, with rare exception (please refer to the Data Availability Statement in the manuscript PDF file). The data should be provided as part of the manuscript or its supporting information, or deposited to a public repository. For example, in addition to summary statistics, the data points behind means, medians and variance measures should be available. If there are restrictions

on publicly sharing data—e.g. participant privacy or use of data from a third party—those must be specified.

Reviewer #1: Yes

Reviewer #2: Yes

4. Is the manuscript presented in an intelligible fashion and written in standard English?

PLOS ONE does not copyedit accepted manuscripts, so the language in submitted articles must be clear, correct, and unambiguous. Any typographical or grammatical errors should be corrected at revision, so please note any specific errors here.

Reviewer #1: Yes

Reviewer #2: Yes

Response to Reviewers' Comments and Questions

Please use the space provided to explain your answers to the questions above. You may also include additional comments for the author, including concerns about dual publication, research ethics, or publication ethics. (Please upload your review as an attachment if it exceeds 20,000 characters)

Reviewer #1: Authors eliminate the IRI, RSI, and RDI by using the hybrid zeroforcing and singular value decomposition (ZF-SVD) beamforming technique based on nullspace projection. Paper is technically sound and well written, i have few major comments:

1: It is important to show the effect on mobility.

Response: Thank you for the suggestion, the relay mobility have been added with an additional figure (8).

2: Some important references are missing: a: NOMA-Based Coordinated Direct and Relay Transmission With a Half-Duplex/ Full-Duplex Relay," in IEEE Transactions on Communications, vol. 68, no. 11, pp. 6750-6760, Nov. 2020, 2:"Heterogeneous Semi-Blind Interference Alignment in Finite-SNR Networks With Fairness Consideration," in IEEE Transactions on Wireless Communications, vol. 19, no. 4, pp. 2472-2488, April 2020.

Response: Thank you for the suggestion, the two references have been added.

Reviewer #2: This manuscript presents a framework for mitigation of the interference in a heterogenous networks where both full-duplex (FD) and half-duplex (HD) relays are exploited. The novel contribution of this work is the consideration of three kinds of interference (inter-relay interference, residual self-interference, and relay-to-destination interference in a multi-cell environment, especially in a heterogenous

network. The authors also solve a joint power allocation optimization problem for the relays and the sources.

In general, the technical contribution of this work is good and the analysis is presented in a good organization. However, I do have some comments to improve the quality of this manuscript:

1. Although the problem considered in this work is novel and comprehensive, the solutions to the considered problem, such as beamforming, zero-forcing, and singular value decomposition, have been well-studied in the previous works. The beamforming designs for the relays and the destinations are quite independent on each other. Therefore, they are not so difficult to derived.

Response: Beamforming have been used in different networks with different criteria. In this work, our joint analysis will not be possible without beamforming technique which simplifies the heterogeneous network analysis that equipped with MIMO antennas.

2. In the system model, the authors consider two adjacent cells, one of which uses half-duplex relay while the other uses full-duplex relay. This is not a typical situation. Why don't they consider two adjacent cells, both of which employ full-duplex relay?

Response: In cellular standards such as 5G and beyond and due to increased connectivity, in order to satisfy different quality-of-service constraints, ideally the network will comprise of half-duplex, full-deplex, IoT , drone devices, and so on. However, most recent research considered two adjacent cells employing either full–duplex or half-duplex relays. Hence, a heterogeneous network that comprised of multicell, HD and FD is much appreciated.

3. Regarding the joint power allocation for source and relay nodes, why is there the total power constraint? These are two independence devices, so each should has its own maximum available power.

Response: In order to optimise the proposed network as a whole system including the sources, relays and destinations, the total power constraint reflects the available power budget, one could easily replace "1" by a desired power budget.

4. The baseline schemes for comparison are the HD and FD schemes without interference or with interference but without interference cancellation. These comparisons are trivial. Please compare the proposed method with related works that the authors have introduced.

Response: The baseline schemes for comparison are the HD and FD schemes without interference or with interference have been derived from the literature, we simplify the names for the convenience, however, few references have added.

5. In the Introduction section (page 3, lines 78 - 79), the authors claim that "by changing the level of self-interference, the FD ergodic capacity becomes similar to HD ergodic capacity". But then in the next sentence, they stated that "the ergodic capacity of FD achieves better performance than HD ergodic capacity". Please justify these sentences.

Response: The references have been clarified

6. In the comments on Figure 3, the authors claim that "With the increase in SNR, the performance of FD without interference cancellation decreases". This is not compatible with the results in Figure 3.

Response: This updated to “With the increase in SNR, the performance of FD without interference cancellation remains flat”

7. In page 11, line 192, what does C^{(M_{S_j}, M_{r_j}) x 1} means?

Response: The sentence is updated as follows “ The definitions of symbol vectors and channel matrices are shown in the following. $\mathbf{x}_S \in \mathbb{C}^{min(M_{Si},M_{ri})\times 1}$, and $\hat{\mathbf{x}}_S \in \mathbb{C}^{min(M_{Sj},M_{rj})\times 1}$ are the transmitted signals from the S node with dimension $min\,(M_{Si}, M_{ri}) \times 1$ and R node with dimension $min\,(M_{Sj}, M_{rj}) \times 1$, respectively”.

8. Page 8, line 148: "(x)^{+} denotes x or 0" --> this is not correct mathemmatically.

Response: The sentence is updated as follows $[x]^+ \triangleq max\{x, 0\}$.

9. There are many typos and grammar errors in the manuscript: - There are some duplication between the Introduction and the section on Related work, for example: "The main hurdle facing relay assisted ..." or "Small cells such as Femto and Pico ..." - Page 3, lines 84-86: "... relay systems Table 1". - Page 3, lines 101-102: "Though it achieves ... was not considered" --> grammar error. - The first paragraph of the subsection "Summary of Contribution" is not clear in meaning. Also, there is a typo: "Table ??". - "moore-penrose pseudoinverse" --> should be "Moore-Penrose ...".

Response: Thank you for pointing out the syntax errors. The duplicating sentences have been removed. Page 3, lines 84-86: "... relay systems Table 1" is changed to “....relay systems as shown in Table 1”. The first paragraph of the subsection "Summary of Contribution" is changed to “Contribution". *"moore-penrose pseudoinverse" --> is changed to "Moore-Penrose ...".*

"Though it achieves ... was not considered" is updated to “...designed based on additional hardware. However, the system did not capture the network heterogeneity.

10. Table 1 is too long, but the readers still cannot see what is the difference between the proposed work and those works in Table 1.

Response: We have revised the final manuscript with the above suggestion and introduced the last row of the table to highlight our contribution.
