# Supplementary material for: Half-duplex and full-duplex interference mitigation in relays assisted heterogeneous network
Source: PLoS One. 2023 Jun 20;18(6):e0286970. doi: 10.1371/journal.pone.0286970 (PMC10281590; doi:10.1371/journal.pone.0286970)
Supplement: Attachment — Submitted filename: Response_to_Reviewers.pdf [file pone.0286970.s002.pdf]

## Manuscript ID PONE-D-22-34295R1

*Firstly, we would like to thank the editor and the reviewers for their efforts in reviewing our manuscript. The comments are useful and constructive. Following the comments, we have made changes to improve the final manuscript. We also respond to individual concerns of the reviewers, where the comments are printed in italic, followed by the responses. In the revised manuscript, all changes are indicated by red colored fonts. The manuscript is edited in order to comply with length policy imposed by PLOS ONE Journal.*

## Reviewers' comments:

*1. If the authors have adequately addressed your comments raised in a previous round of review and you feel that this manuscript is now acceptable for publication, you may indicate that here to bypass the "Comments to the Author" section, enter your conflict of interest statement in the "Confidential to Editor" section, and submit your "Accept" recommendation.*

*Reviewer #1: All comments have been addressed*

*Reviewer #2: (No Response)*

*2. Is the manuscript technically sound, and do the data support the conclusions?*

*The manuscript must describe a technically sound piece of scientific research with data that supports the conclusions. Experiments must have been conducted rigorously, with appropriate controls, replication, and sample sizes. The conclusions must be drawn appropriately based on the data presented.*

*Reviewer #1: Yes*

*Reviewer #2: Yes*

*3. Has the statistical analysis been performed appropriately and rigorously?*

*Reviewer #1: Yes*

*Reviewer #2: Yes*

*4. Have the authors made all data underlying the findings in their manuscript fully available?*

*The PLOS Data policy requires authors to make all data underlying the findings described in their manuscript fully available without restriction, with rare exception (please refer to the Data Availability Statement in the manuscript PDF file). The data should be provided as part of the manuscript or its supporting information, or deposited to a public repository. For example, in addition to summary statistics, the data points behind means, medians and variance measures should be available. If there are restrictions*

*on publicly sharing data—e.g. participant privacy or use of data from a third party—those must be specified.*

*Reviewer #1: Yes*

*Reviewer #2: Yes*

*5. Is the manuscript presented in an intelligible fashion and written in standard English?*

*PLOS ONE does not copyedit accepted manuscripts, so the language in submitted articles must be clear, correct, and unambiguous. Any typographical or grammatical errors should be corrected at revision, so please note any specific errors here.*

*Reviewer #1: Yes*

*Reviewer #2: Yes*

## 6. Review Comments to the Author

*Please use the space provided to explain your answers to the questions above. You may also include additional comments for the author, including concerns about dual publication, research ethics, or publication ethics. (Please upload your review as an attachment if it exceeds 20,000 characters)*

*Reviewer #1: Authors have addressed all my comment's, not more comments. Authors have addressed all my comment's, not more comments. Authors have addressed all my comment's, not more comments.*

Response: Thank you very much

*Reviewer #2: In this revised manuscript, the authors have made some appropriate changes to improve the presentation of their work. However, some of technical concerns that I have commented in the previous review have not been responded seriously.*

*1. Regarding the comment on the assumption that the adjacent cell uses half-duplex transmission instead of full-duplex, please state clearly in the manuscript the practical situations that this work can be applied. This should be done to show a solid motivation for their work.*

Response: We have revised the final manuscript with the above suggestion (lines 161 to 168).

*2. Regarding the question on the constrain on the total power constraint, it seems that the authors misunderstood my comment. I don't care what the values the authors set for the total power constraint (1 or any). My main concern is that any device should have their own power constraint, which does not depend on the power consumption of other devices. In your model, does the relay need to contact the base station to know about the maximum power that it (the relay) can transmit? Please clarify this setup by solid argument, not by references (because this should be specific to your model).*

Response: We have revised the final manuscript with the above clarifications (lines 327 to 336).

*3. In response to the concern about the claim "by changing the level of self-interference, the FD ergodic capacity becomes like HD ergodic capacity", then "Finally, the ergodic capacity of FD achieves better performance than HD ergodic capacity", the authors referred to two previous references but actually, they were not related. So, please elaborate on those claims or rewrite the sentences to make it easier to follow.*

*In general, I expect more serious response from the authors on the technical points, not just 2-3 sentences and without any changes in the manuscript.*

Response: We revised the final manuscript and highlighted the above suggestions (lines 77 to 83). Thank you for taking the time to improve the manuscript; we eagerly await your feedback.
